# A novel flow cytometry panel to identify prognostic markers for steroid-sensitive forms of idiopathic nephrotic syndrome in childhood

**DOI:** 10.3389/fimmu.2024.1379924

**Published:** 2024-04-02

**Authors:** Martina Riganati, Federica Zotta, Annalisa Candino, Ester Conversano, Antonio Gargiulo, Marco Scarsella, Anna Lo Russo, Chiara Bettini, Francesco Emma, Marina Vivarelli, Manuela Colucci

**Affiliations:** ^1^ Laboratory of Nephrology, Bambino Gesù Children’s Hospital Scientific Institute for Research and Health Care (IRCCS), Rome, Italy; ^2^ Division of Nephrology, Bambino Gesù Children’s Hospital Scientific Institute for Research and Health Care (IRCCS), Rome, Italy; ^3^ Research Laboratories, Bambino Gesù Children’s Hospital Scientific Institute for Research and Health Care (IRCCS), Rome, Italy

**Keywords:** idiopathic nephrotic syndrome, B cell, T cell, lymphocyte profile, prognostic markers

## Abstract

**Introduction:**

The clinical evolution of steroid-sensitive forms of pediatric idiopathic nephrotic syndrome (INS) is highly heterogeneous following the standard treatment with prednisone. To date, no prognostic marker has been identified to predict the severity of the disease course starting from the first episode.

**Methods:**

In this monocentric prospective cohort study we set up a reproducible and standardized flow cytometry panel using two sample tubes (one for B-cell and one for T-cell subsets) to extensively characterized the lymphocyte repertoire of INS pediatric patients. A total of 44 children with INS at disease onset were enrolled, sampled before and 3 months after standard induction therapy with prednisone and followed for 12 months to correctly classify their disease based on relapses. Age-matched controls with non immune-mediated renal diseases or with urological disorders were also enrolled. Demographical, clinical, laboratory and immunosuppressive treatment data were registered.

**Results:**

We found that children with INS at disease onset had significantly higher circulating levels of total CD19^+^ and specific B-cell subsets (transitional, mature-naïve, plasmablasts/plasmacells, CD19^+^CD27^+^, unswitched, switched and atypical memory B cells) and reduced circulating levels of Tregs, when compared to age-matched controls. Prednisone therapy restored most B- and T-cell alterations. When patients were subdivided based on disease relapse, relapsing patients had significantly more transitional, CD19^+^CD27^+^ memory and in particular unswitched memory B cells at disease onset, which were predictive of a higher risk of relapse in steroid-sensitive patients by logistic regression analysis, irrespective of age. In accordance, B-cell dysregulations resulted mainly associated with steroid-dependence when patients were stratified in different disease severity forms. Of note, Treg levels were reduced independently from the disease subgroup and were not completely normalized by prednisone treatment.

**Conclusion:**

We have set up a novel, reproducible, disease-specific flow cytometry panel that allows a comprehensive characterization of circulating lymphocytes. We found that, at disease onset, relapsing patients had significantly more transitional, CD19^+^CD27^+^ memory and unswitched memory B cells and those who are at higher risk of relapse had increased circulating levels of unswitched memory B cells, independently of age. This approach can allow prediction of clinical evolution, monitoring of immunosuppression and tailored treatment in different forms of INS.

## Introduction

Idiopathic nephrotic syndrome (INS) is the most frequent glomerular disease in childhood, characterized by a damage of the glomerular filtration barrier leading to loss of protein in the urine, hypoalbuminemia and oedema (1).

At first presentation, patients receive a standard course of oral prednisone, to which the majority of children respond within 4 weeks and are therefore defined as steroid-sensitive (SSNS) (1). The clinical evolution of SSNS is highly heterogeneous, ranging from non-relapsing or infrequently relapsing forms (NRNS/IRNS), to frequently relapsing forms (FRNS), to patients that are steroid-dependent (SDNS) (1). Approximately 60% of patients require steroid-sparing immunosuppressive agents and 5-42% of patients continue to experience relapses in adulthood (1). A minority of patients, 10-15%, are steroid-resistant (SRNS) and at risk of progressing to kidney failure (1, 2). Currently, no prognostic markers allow an accurate prediction of clinical evolution at disease onset. Therefore, the initial treatment protocol is standardized for all patients (1).

For nearly 50 years, INS has been considered a renal manifestation of a systemic T-cell dysregulation (3). In the past decade, the therapeutic efficacy of anti-CD20 B-cell-depleting monoclonal antibodies, mainly rituximab, has implicated B cells in the pathogenesis of the disease (4, 5). In pediatric patients with SSNS, alterations in B-cell homeostasis that can be observed at disease onset, before starting any treatment, have been reported by our group and by others (as reviewed in (6)). To date, several studies have attempted to discriminate SSNS from SRNS forms at disease onset using flow cytometry (7–9). However, comprehensive flow cytometry profiling techniques that allow a complete characterization of lymphocyte subpopulations are not currently available.

To this end, we have set up a reproducible and standardized flow cytometry panel that allows detailed characterization of the lymphocyte subpopulations, in order to define a disease-specific B- and T-cell “signature”. This tool may allow prediction of clinical evolution, treatment monitoring, and tailored therapeutic approaches to different forms of the disease.

## Materials and methods

### Patient selection

We conducted a prospective cohort study including all children with INS who presented at disease onset at the Bambino Gesù Children’s Hospital - IRCCS from July 2018 to December 2023. The local institutional review board approved the study, and written informed consent was provided by the participants’ legal guardians/next of kin. The study was performed in compliance with the declaration of Helsinki. Clinical definitions of nephrotic syndrome (IRNS, FRNS, SDNS, SSNS, and SRNS), remission and relapse are listed in **Supplementary Table S1** (1). Inclusion criteria were INS with age at onset below 18 years. Exclusion criteria were congenital or genetic forms of NS, secondary forms of NS, chronic infections, previous treatment with immunosuppressive drugs (excluding low dose steroids for periods <3 months). All patient samples were obtained at disease onset, before starting oral prednisone. The initial therapy was as follows: oral prednisone 60 mg/m^2^/daily for 6 weeks followed by 40 mg/m^2^/every other day for 6 weeks. All patients were followed for 12 months, in order to correctly classify their disease based on relapses. Per internal protocol, renal biopsy was performed only in patients aged ≤1 years or ≥12 years old at disease onset, if clinical finding suggestive of other glomerular disorders were present, or in the absence of response to prednisone therapy after 4 weeks. Age-matched controls with non immune-mediated renal diseases or with urological disorders were also enrolled. Exclusion criteria for control patients included chronic renal failure (estimated glomerular filtration rate (eGFR) < 60 ml/min/1.73 m^2^), chronic infections, previous treatment with immunosuppressive drugs (excluding low dose steroids for periods <3 months). Demographical, clinical, laboratory and immunosuppressive treatment data were registered for all patients during the follow-up.

### Laboratory data

Whole blood cell count, serum creatinine, serum albumin, serum total proteins, serum C reactive protein, serum cholesterol and the urine protein-to-creatinine-ratio were recorded. Nasopharyngeal aspirates were obtained if patients presented with respiratory symptoms at onset. CRP > 0.5 mg/dl or positivity at the nasopharyngeal aspirate were considered signs of bacterial or viral infection.

### Cell collection

Peripheral blood mononuclear cells (PBMCs) were isolated by Pancoll human (Pan Biotech) density-gradient centrifugation after blood collection in EDTA tubes at disease onset and if available after 3 months. Cells were frozen in bovine serum with 10% DMSO and stored in liquid nitrogen until the flow cytometry analysis.

### Flow cytometry analysis

**Figure 1:**
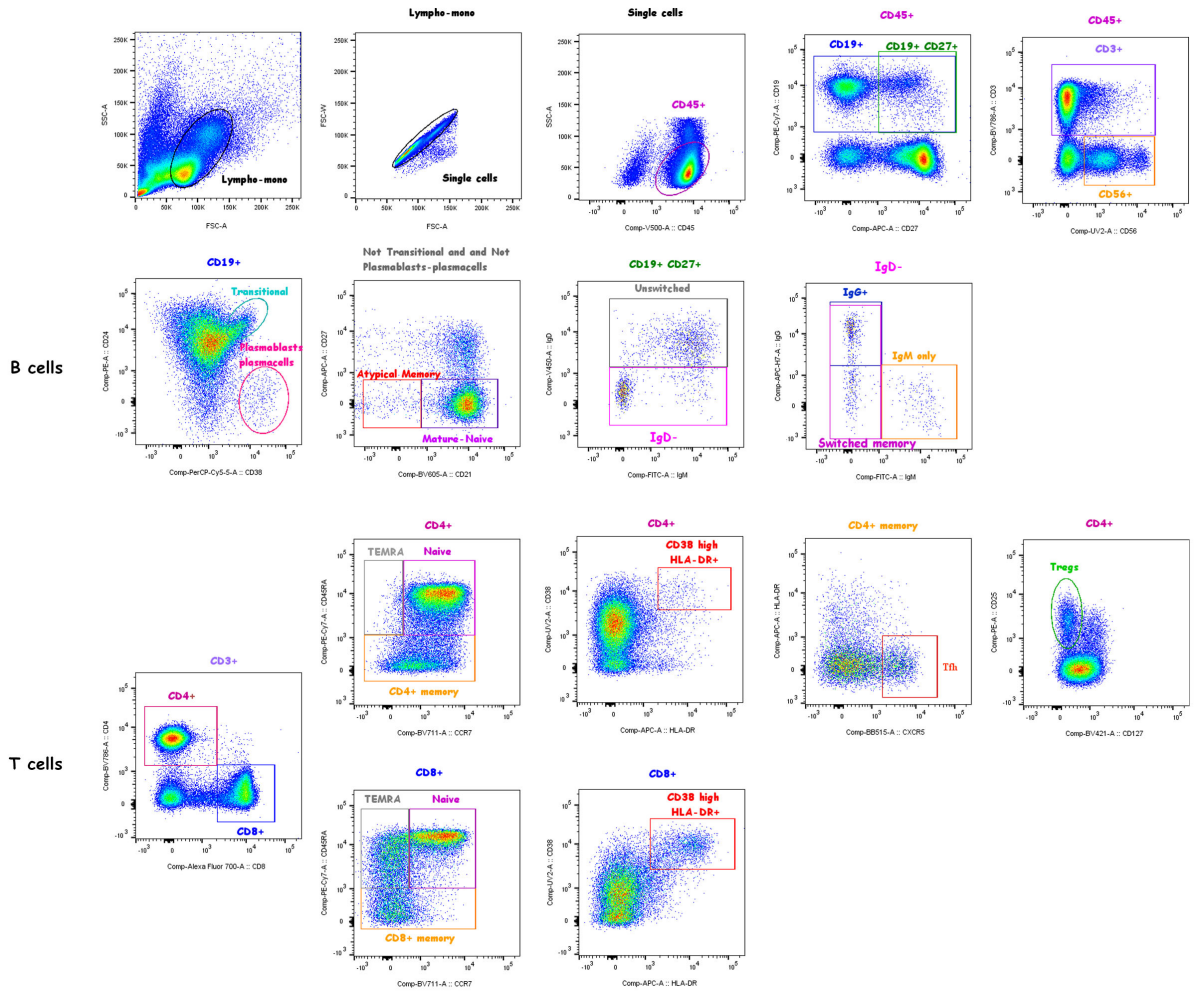
Gating strategy. Live CD45^+^ lymphocytes were identified based on the FSC/SSC lympho-monocyte and singlet gates. Subsets of gated total CD19^+^ B cells were identified based on the expression of surface markers as follows: transitional (CD38^high^CD24^high^), plasmablasts/plasmacells (CD38^high^CD24^-^), mature-naïve (CD21^+^CD27^-^) and atypical memory (CD21^-^CD27^-^) identified in the “Not transitional-not plasmablasts/plasamcells” population; memory B cells were defined as CD19^+^CD27^+^ cells and memory subclasses were defined as unswitched memory (IgM^+^IgD^+^, also known as IgM memory), switched memory (IgM^-^IgD^-^), IgM only memory (IgM^+^IgD^-^IgG^-^), IgG^+^ switched memory (IgM^-^IgD^-^IgG^+^) B cells. Subsets of gated total CD3^+^ T cells were identified based on the expression of surface markers as follows: CD4^+^ or CD8^+^ T cells were identified as naïve (CD45RA^+^CCR7^+^), memory (CD45RA^-^), TEMRA (CD45RA^+^CCR7^-^) and highly activated (CD38^high^HLA-DR^+^) subsets; CD4^+^ Tregs (CD25^high^CD127^-^) and CD4^+^ Tfh cells (CXCR5^+^HLA-DR^-^) were also identified. NK cells were identified as CD56^+^ cells in CD45^+^ live gated lymphocytes.

To distinguish lymphocyte subpopulations, thawed PBMCs (1x10^6^ cells/sample tube) were stained with fluorochrome-conjugated monoclonal Abs directed against the following surface markers in two different tubes as follows: Tube 1: CD3, CD19, CD21, CD24, CD27, CD38, CD45, CD56, IgD, IgM and IgG (BD Biosciences); Tube 2: CD3, CD4, CD8, CD45RA, CCR7, CD25, CD127, CXCR5, CD38, HLA-DR (**Supplementary Table S2**). Stained cells were analyzed by a FACS BD LSRFortessa (BD Biosciences) (see **Figure 1** for gating strategy). Analyses were performed using the program FlowJo, version 10 (Tree Star, Ashland, OR).

### Statistical analyses

Continuous data are expressed as mean ± standard deviation if they passed the normality test (Shapiro–Wilk test), or median and interquartile range otherwise. Categorical data are represented as numbers and percentages. Comparison between INS patients at onset and controls and between relapsing and non-relapsing patients were analyzed by unpaired t test if normally distributed, or nonparametric Mann–Whitney U test. Differences between subgroups were analyzed using a nonparametric Kruskal–Wallis test and, if significant, pairwise comparisons were evaluated by the Dunn’s test. Correlations were tested using the Spearman’s rank order test. The association between several parameters at onset and the risk of relapse during the 12-month follow-up was evaluated by logistic regression model. Covariates were included in multivariable modelling whether they reached a significant p value in univariate analysis. The predictive role of lymphocyte subsets significantly associated with the risk of relapse by multivariable logistic regression was analyzed by receiver operating characteristic (ROC) analysis, and by the Kaplan–Meier method (log-rank test). All p values are two sided and considered statistically significant with p-value <0.05. Analyses were performed using Graphpad Prism 9.0.

## Results

### Study patients

Overall, 58 pediatric patients were initially enrolled in the study. Fourteen patients were subsequently excluded due to having genetic forms of NS (1 patient), NS secondary to other diseases (2 membranous nephropathy, 1 C3 glomerulopathy), or for having started prednisone before sampling (10 patients) (**Supplementary Figure S1**). Overall, 44 patients (22 males) were included in the study and 44 age-matched subjects were used as controls (26 males) (13 with congenital anomalies of the kidneys and urinary tract (CAKUT), 8 with kidney cysts, 5 with microhematuria/GBM pathology, 11 with nephrocalcinosis, 7 with a previous history of urinary tract infection). **Table 1** and **Supplementary Table S3** summarize demographic, clinical and laboratory characteristics at the time of sampling.

**Table 1:** Patient characteristics.

Parameters	Unit	CTRL	INS Onset	INS Onset +3 months
		(n=44)	(n=44)	(n=28)
**Demographic**				
**Age (years)**	Years	4.8 [3 - 6.7]	4.1 [2.9 - 6.9]	–
**Male**	N(%)	26(59)	22 (50)	–
**Ethnicity (White)**	N(%)	43 (97.7)	35 (79.5)	–
**Clinical**				
**eGFR**	ml/min/m2	129 [104 - 148]	149.1*** [117.3 - 193]	139 [129.9 - 155.5]
**serum protein**	g/dl	–	4.4 [4 - 5.5]	6.3### [5.7 - 6.8]
**serum albumin**	g/dl	–	2.3 [2 - 3.7]	4.4### [4 - 4.5]
**serum Cholesterol**	mg/dl	–	352 [288 - 538]	168### [136 - 226]
**urinary Protein/creatinine**	mg/mg	0.16 [0.14 - 0.218]	11.45*** [4.995 - 21.79]	0.23### [0.14 - 0.37]
**C reactive Protein**	mg/dl	0.03 [0.03 - 0.2775]	0.03 [0.03 - 0.18]	0.07 [0.03 - 0.12]
**Signs of infection**	N(%)	–	10 (23)	–
**Disease characteristics**				
**Disease forms** **(IRNS-NRNS/FRNS/SDNS/SRNS)**	N	–	15/4/21/4	9/4/12/3
**Active phase/disease form**	N(%)	–	15(100)/4(100)/21(100)/4(100)	0(0)/0(0)/4(33)/2(67)
**Time to remission**	days	–	9.5 [7.0-14.5]	–
**Relapsing patients during 12-month follow-up**	N(%)	–	28 (64)	–
**Time to 1^st^ relapse after remission**	days	–	132.0 [88.3-365]	–
**Immunosupressive treatment/disease form**				
**prednisone alone**	N(%)	–	–	1(11)/0(0)/3(25)/0(0)
**prednisone + mycophenolate mofetil**	N(%)	–	–	2(22)/0(0)/0(0)/0(0)
**prednisone + calcineurin inhibitors**	N(%)	–	–	0(0)/0(0)/3(25)/3(100)
**Lymphocyte subsets**				
**CD19+ B cells**	% of CD45+	18 [13.58 - 19.25]	23.45** [16.9 - 33.2]	10.3***,### [7.753 - 12.75]
**CD3+ T cells**	% of CD45+	69.6 [65.17 - 76.52]	63.6** [52.11 - 71.37]	68.54 [57.95 - 75.03]
**CD56+ NK cells**	% of CD45+	12.25 [5.788 - 17.28]	8.07** [4.7 - 11.05]	15.7*,### [12.28 - 21.7]
**Transitional B cells**	% of CD45+	1.63 [1.25 - 2.383]	3.17** [1.883 - 4.448]	1.18### [0.245 - 2.06]
**Mature-naive B cells**	% of CD45+	9.22 [7.42 - 12.75]	13,76** [8.605 - 18.36]	4,47***,### [3.213 - 5.55]
**Plasmablasts/plasmacells**	% of CD45+	0.172 [0.115 - 0.3218]	0,3** [0.1718 - 0.565]	0.304 [0.09325 - 0.5138]
**Atypical memory B cells**	% of CD45+	0.29 [0.21 - 0.43]	0,37* [0.2375 - 0.615]	0,21### [0.13 - 0.3675]
**CD19+CD27+ memory B cells**	% of CD45+	2.77 [2.058 - 3.83]	3,865** [2.555 - 5.213]	2,57## [1.84 - 3.44]
**Unswitched memory B cells**	% of CD45+	1.58 [1.015 - 1.87]	1,835** [1.373 - 2.75]	1,305### [0.8025 - 1.71]
**Switched memory B cells**	% of CD45+	1.19 [0.74 - 1.785]	1,505* [0.95 - 2.55]	1.12 [0.77 - 1.778]
**IgM only memory B cells**	% of CD45+	0.15 [0.1125 - 0.27]	0.195 [0.14 - 0.37]	0,145## [0.08 - 0.1975]
**IgG+ switched memory B cells**	% of CD45+	0.82 [0.455 - 1.113]	0,925* [0.5875 - 1.518]	0.71 [0.4825 - 1.128]
**CD4+ T cells**	% of CD45+	36.69 [26.9 - 41.9]	32,26* [23.51 - 37.97]	28,46** [22.74 - 35.98]
**CD4+ TEMRA**	% of CD45+	0.47 [0.2775 - 1.878]	0.79 [0.385 - 1.185]	1.11 [0.785 - 1.435]
**CD4+ Naive T cells**	% of CD45+	24.68 [16.6 - 29.78]	18.69 [13.13 - 32.96]	17,84* [12.97 - 22.33]
**CD4+ memory T cells**	% of CD45+	8.955 [6.843 - 13.15]	7.15 [4.595 - 9.525]	8.71 [6.418 - 10.57]
**CD4+CD38highHLA-DR+**	% of CD45+	0.07 [0.06 - 0.1275]	0.065 [0.05 - 0.0975]	0.07 [0.0475 - 0.1025]
**Tfh cells**	% of CD45+	1.205 [0.685 - 1.733]	1.02 [0.535 - 1.748]	1.02 [0.77 - 1.66]
**Treg cells**	% of CD45+	2.265 [1.708 - 3.063]	1,62*** [1.113 - 2.28]	1,69* [1.233 - 2.44]
**CD8+ T cells**	% of CD45+	21.54 [18.22 - 28.74]	23.68 [20.28 - 27.38]	26,57* [21.24 - 34.5]
**CD8+ TEMRA**	% of CD45+	7.705 [5.913 - 9.105]	4,47** [3.41 - 7.705]	7,165# [4.968 - 11.84]
**CD8+ Naive T cells**	% of CD45+	10.77 [8.435 - 13.46]	12,81* [10.92 - 17.45]	13,05* [11.07 - 18.04]
**CD8+ memory T cells**	% of CD45+	3.02 [1.87 - 4.78]	2.65 [1.78 - 5.125]	3.56 [2.355 - 5.83]
**CD8+CD38highHLA-DR+**	% of CD45+	0.15 [0.08 - 0.265]	0,26* [0.1225 - 0.5075]	0.18 [0.1075 - 0.5225]

*, **, *** significant differences vs CTRL.

##, ### significant differences vs INS at onset.

No significant difference was observed in the demographical characteristics when comparing INS patients to control patients (**Table 1**). A significantly lower median age at onset was observed in SDNS patients compared to NRNS/IRNS patients (**Supplementary Table S3**). Estimated (e)GFR and proteinuria were significantly higher in INS patients at onset compared to controls (**Table 1**). Signs of intercurrent infection were observed in 10 INS patients at disease onset, as determined by CRP > 0.5 mg/dl or positivity at the nasopharyngeal aspirate (**Table 1**). Signs of infection were not evaluated in control patients, since no nasopharyngeal aspirate was performed in this group. CRP levels were comparable between patients and controls (**Table 1**). No significant differences were observed in laboratory analyses between subgroups of INS patients, except for lower eGFR in NRNS/IRNS compared to SDNS (**Supplementary Table S3**). After beginning prednisone therapy, 4 patients did not achieve remission within 4 weeks and were considered SRNS. The median time to achieve remission in SSNS was 9.5 [7.0-14.5] days. At 12 months, 28 SSNS patients relapsed after a median time of 132.0 [88.3-365] days from remission. Overall, 15 patients were classified as NRNS/IRNS, 4 patients as FRNS, 21 patients as SDNS, and 4 patients as SRNS, as defined in **Supplementary Table S1**. A second sample was obtained after a mean time of 3.1 ± 0.5 months in 28 INS patients, including 9 patients with NRNS/IRNS, 4 patients with FRNS, 12 patients with SDNS and 3 patients with SRNS: most patients were in complete remission, except for 4 SDNS and 2 SRNS patients who were in active phase of disease. At the time of the second sample collection, 12 patients were receiving immunosuppressive prednisone, which was associated with mycophenolate mofetil or cyclosporin A in 8 patients (**Table 1**).

### Lymphocyte profile

**Figure 2:**
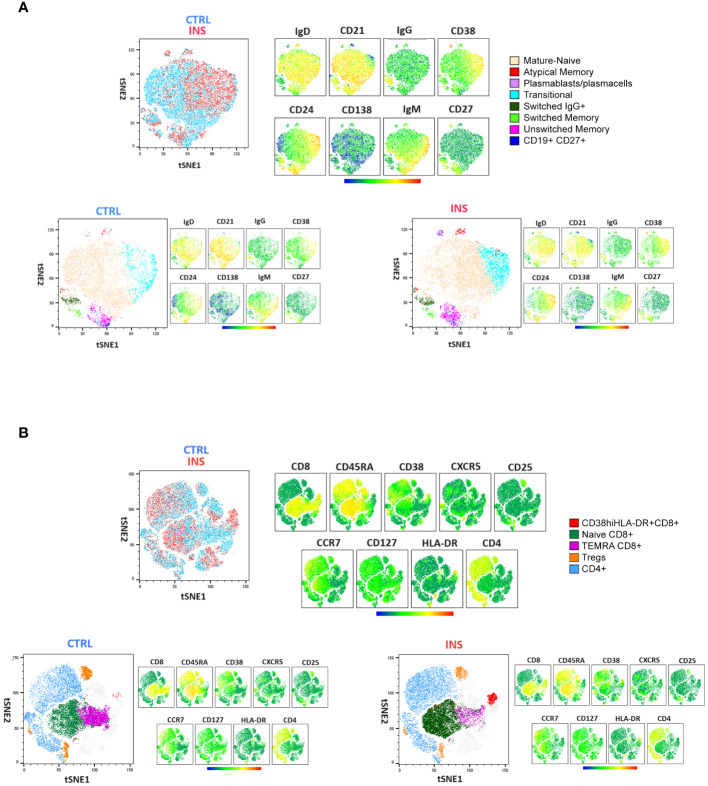
Comprehensive lymphocyte profile representation of idiopathic nephrotic syndrome patients and age-matched controls. tSNE analysis followed by clustering of flow cytometry data defined by the manual gating strategy in **Figure 1**. Surface **(A)** B-cell and **(B)** T-cell marker distribution in a single idiopathic nephrotic syndrome pediatric patient (INS) and a single age-matched control (CTRL) are shown. tSNE plots were represented as merged (upper panels) or separated (lower panels). The specific identified B-cell and T-cell subsets are indicated by colors. Relative antigen expression was visualized by analysis logarithmic stochastic Heat Map (from blue to red).

The distribution of the different lymphocyte subsets was evaluated in INS patients at onset and after 3 months of prednisone therapy and compared to age-matched controls (**Table 1**). At onset, significantly higher median circulating levels of total CD19^+^ B cells associated with significantly lower median levels of total CD3^+^ T cells and CD56^+^ NK cells were found compared to controls (**Table 1**). Most B-cell subsets (transitional, mature-naïve, plasmablasts/plasmacells, and CD19^+^CD27^+^, unswitched, switched and atypical memory B cells) were significantly higher in INS patients at onset compared to controls (**Table 1**, **Figure 2A**). In contrast, fewer T-cell dysregulations were found in INS patients at onset compared to controls, with a significant reduction of total CD4^+^ T cells, CD4^+^ Tregs and CD8^+^ TEMRA cells and a significant increase of CD8^+^ naïve T cells and highly activated CD8^+^CD38^high^HLA-DR^+^ T cells, with no difference in the amount of Tfh cells (**Table 1**, **Figure 2B**). No significant correlation was found between signs of infection and each lymphocyte subset, except for a direct correlation with switched memory B cells (r= 0.308, p= 0.042) in INS patients at onset. The completion of the standard course of prednisone therapy normalized or significantly decreased most B- and T-cell alterations (**Table 1**). In particular, total CD19^+^ and mature-naïve B cells were strongly and significantly reduced and CD56^+^ NK cells were significantly increased after 3 months of prednisone therapy compared to both INS at onset and age-matched controls (**Table 1**). In addition, transitional, plasmablasts/plasmacells, CD19^+^CD27^+^, unswitched, switched and atypical memory B cells, CD8^+^ TEMRA and highly activated CD8^+^CD38^high^HLA-DR^+^ T cells were normalized after prednisone therapy, whilst no significant effect was found on the amount of CD4^+^ Tregs and CD8^+^ Naïve T cells (**Table 1**).

**Figure 3:**
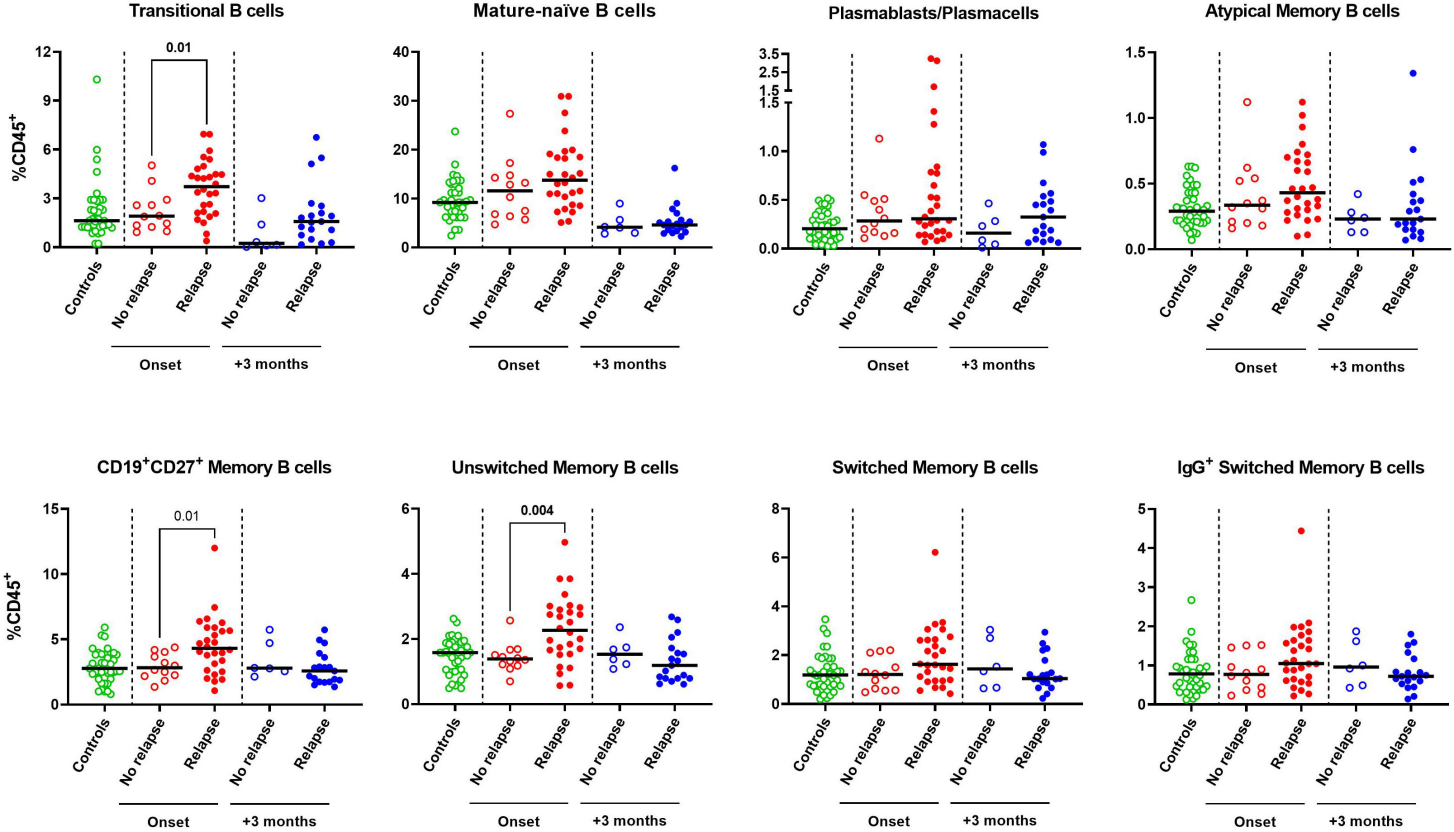
B-cell subset profile of steroid-sensitive nephrotic syndrome pediatric patients who experienced or not a relapse event during a 12-month follow-up. Circulating levels of transitional, mature-naive, plasmablasts/plasmacells, atypical memory, total CD19^+^CD27^+^ memory, unswitched memory, switched memory and IgG^+^ switched memory were compared between patients who relapsed or not during a 12-month follow-up as determined at onset (red dots, n=28 vs n=12) or after 3 months of prednisone therapy (blue dots, n=22 vs n=6). Age-matched controls were also represented (green dots, n=40). B-cell subsets were expressed as percentages of total CD45^+^ lymphocytes. Horizontal lines indicate the medians. Differences between groups were compared using unpaired Mann-Whitney U test.

**Figure 4:**
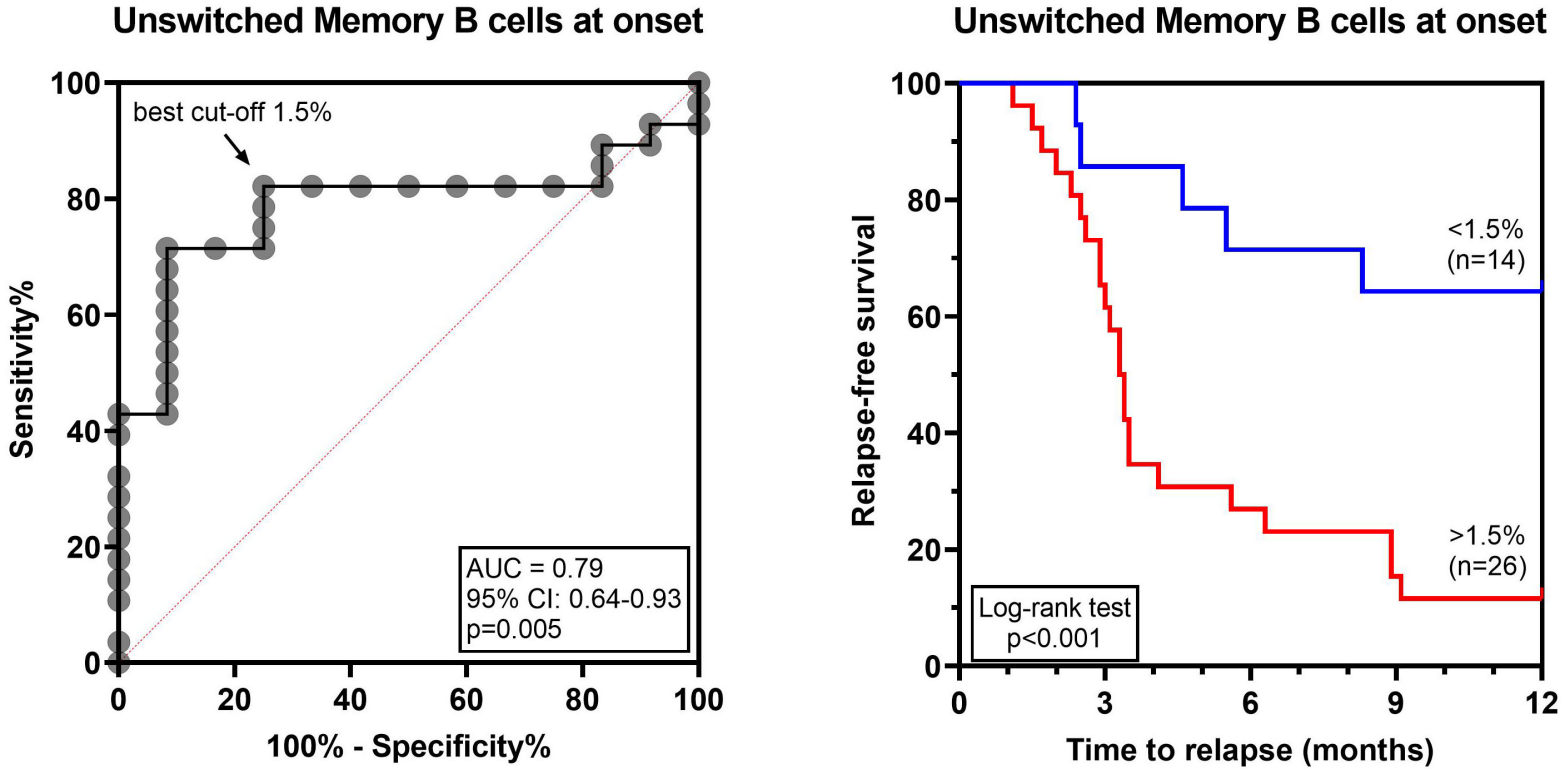
Unswitched memory B-cell levels at disease onset are predictive of relapse in INS pediatric patients. ROC curve analyzing unswitched memory B-cell levels and the risk of relapse within 12 months of follow-up. The arrow indicates the best cut-off for unswitched memory B cells 1.5%. Relapse-free survival was compared between patients with unswitched memory B-cell levels above (red line) or below (blue line) the cut-off by log-rank test. AUC, area under the curve.

To determine whether the lymphocyte profile at disease onset could predict the risk of relapse, the 40 SSNS were subdivided as relapsers and non-relapsers during the 12-month follow-up. No significant difference was found in total CD19^+^ B cells, CD3^+^ T cell and CD56^+^ NK cells (**Supplementary Figure S2**). Among the different B-cell and T-cell susbets, we found that, at onset, relapsing patients had significantly higher median levels of transitional B cells (3.7% vs 1.9%, p=0.01), CD19^+^CD27^+^ memory B cells (4.3% vs 2.8%, p= 0.01) and of unswitched memory B-cell subsets (2.3% vs 1.4%, p=0.004) compared to non-relapsing patients (**Figure 3**). In particular, CD19^+^CD27^+^ and unswitched memory B cells were significantly associated with the risk of relapse by logistic regression (OR, 1.9 and 4.6, respectively; p<0.05) and unswitched memory B cells only retained this significant association also when adjusted for age (OR, 3.6; p<0.05). The best threshold for discriminating relapsing from non-relapsing patients identified by ROC analysis was 1.5% of unswitched memory B-cell levels (AUC= 0.79, p= 0.005, **Figure 4**). No further significant difference was found in each other analyzed lymphocyte subset (**Figure 3**, **Supplementary Figures S3**, **S4**). In addition, no significant difference was found among relapsers and non-relapsers after 3 months of prednisone therapy (**Figure 3**, **Supplementary Figures S2**-**S4**).

INS patients were subsequently subdivided based on the clinical course of their disease during the follow-up and each subgroup (NRNS/IRNS, FRNS, SDNS, SRNS) was compared with its own age-matched control subgroup (**Supplementary Figures S5**, **S6**). SDNS patients were those with the most relevant B- and T-cell dysregulations (**Supplementary Figures S5**, **S6**). SDNS patients also had significantly higher levels of transitional, mature-naïve and unswitched memory B-cell subsets when compared to NRNS/IRNS, whilst no significant difference was found in any of the analyzed T-cell subsets or in CD56^+^ NK cells (**Supplementary Table S3**). Of note, we failed to find a lymphocyte subset discriminating SDNS from FRNS, also due to the low number of patients classified as FRNS.

## Discussion

The aim of this study was to develop a reproducible comprehensive flow cytometry panel to define specific B- and/or T-cell “signatures” in patients with INS. To validate this approach, we have used this technology in children at disease onset, to determine whether specific lymphocyte subpopulations before starting prednisone treatment correlate with median-term prognosis - i.e. with relapse at 12 months - and after 3 months of standard immunosuppressive induction therapy with prednisone to assess the effects of this treatment on lymphocyte profiles. The ultimate goal of this approach is to tailor therapy to individual disease severity and to assess sensitivity to immunosuppressive drugs. Our results show that extended characterization of lymphocyte subpopulations can be performed using only two sample tubes (on for the B-cell and one for the T-cell repertoire). This methodology requires a very limited number of cells, a crucial advantage in a pediatric setting. Antibodies that were used were directed only against surface antigens, as opposed to intracellular epitopes, in order to minimize laborious and less reproducible procedures. We analyzed only cryopreserved cells after thawing not requiring stimulation, which is particularly advantageous as it allows sample shipment to Institutions equipped with the appropriate instruments, facilitating multicentric studies.

Using this methodology, we observed that children with INS at disease onset had higher circulating levels of CD19^+^ B cells when compared to age-matched controls, before starting immunosuppressive therapies. The sub-analysis of B-cell subpopulations showed significant increase in transitional, mature-naïve, plasmablasts/plasmacells, CD19^+^CD27^+^, unswitched, switched and atypical memory B cells. When patients were subdivided based on disease relapse during a 12-month follow-up, relapsing patients had significantly more transitional, CD19^+^CD27^+^ memory and unswitched memory B cells at disease onset. Increased unswitched memory B-cell levels were also predictive of a higher risk of relapse in SSNS patients by logistic regression analysis, independently of age. In accordance, B-cell dysregulations resulted mainly associated with SDNS. These results are in agreement with previous studies reporting dysregulations of several sub-population of B-cells in children with INS (6). In most reports, increased circulating levels of total CD19^+^ B cells have been observed before therapy, which were reduced after prednisone treatment (7, 8, 10–12). Among different B-cell subsets, an expansion of transitional and in particular of memory B cells has been reported more frequently, in both pediatric and adult patients (7, 9, 11, 12), together with increased mature-naïve B cells in some studies (8, 13). A recent characterization of the B-cell transcriptional profile in INS patients in active phase has shown increased expression of genes associated with antibody-secreting B cells, atypical memory B cells, and total memory B cells, both of switched and unswitched “MZ-like” subtype (14). The novel comprehensive flow cytometry panel described in the current study confirmed these observations and increased the sensitivity of our initial methodological approach which was effective in identifying the expansion of transitional, total memory and switched memory B cells in SSNS patients at disease onset but failed to assess accurately mature-naïve, plasmablasts/plasmacells and unswitched memory B cells, probably due to a different gating strategy (11, 15).

In contrast to B-cell dysregulations, we observed modest changes in CD56^+^ NK cell and T-cell subsets. A significant reduction in Treg cells was also observed, as previously described (16, 17).

Stratification of patients in different disease severity classes was limited by the number of patients, which underpowered comparisons. Nonetheless, we observed lower Treg levels compared to control independently from the disease subgroup (i.e. NRNS/IRNS or SDNS), suggesting that lower levels of Tregs are not associated with disease severity, whereas CD8^+^CD38^high^HLA-DR^+^ T cells were more often increased in SDNS patients. A dysregulation of this highly activated population of T cells has already been reported in SSNS pediatric patients (8) and its role in the pathogenesis of the disease is under investigation. Expansion of this cell subset can also be observed in hyper-inflammatory conditions such as hemophagocytic lymphohistiocytosis (18) or acute viral infections (19).

As expected, prednisone therapy normalized most B- and T-cell subsets, although circulating Tregs were not completely normalized after prednisone therapy, contrary to previous reports, although comparisons are limited by differences in the analyzed time points (17, 20). With this methodology, which allows more frequent monitoring without excessive blood amount, we hope in the future to better study the dynamics of Treg levels in pediatric INS.

The goal of this study was primarily methodological. The analysis of patients with INS served primarily as a mean to validate this technology and is therefore limited in its interpretation by the relatively low number of patients, although samples at disease onset are not easy to collect. Nonetheless, rigorous inclusion criteria and a uniform follow-up at 12-months strengthen the analysis and allowed to divide the population in “early” relapsers and patients that had not relapsed at 12 months. With this categorization of patients, differences in B-cell populations at disease onset, even after correcting for patient age, could be observed. Results were also strengthened by the inclusion of an age-matched control population and by repeat analyses after immunosuppressive therapy. Another limitation of this study is that our approach did not allow characterization of Th1/Th2/Th17 dysregulations, which have been demonstrated to sustain active disease state in INS (16, 17, 21, 22). Currently, the characterization of these Th cell subsets can be performed only by intracellular cytokine staining, which requires more complex sample processing that can compromise the reproducibility of the results (23), or by the analysis of surface chemokine receptor staining, which is affected by cryopreservation ((23) and our unpublished observations).

In conclusion, we have set up a novel, reproducible, disease-specific flow cytometry panel that allows a comprehensive characterization of circulating B- and T-cells. With this method we found that relapsing patients had significantly more transitional, CD19^+^CD27^+^ memory and unswitched memory B cells at disease onset and identified increased unswitched memory B cell levels as predictors of a higher risk of relapse at 12 months, independently of age. If confirmed, these results may assist in developing different treatment strategies according to the B-cell expression profile, restricting more aggressive immunosuppressive therapy to selected severe patients. They may also provide clues to identify novel and more tailored targets of therapy.

## Data availability statement

The raw data supporting the conclusions of this article will be made available by the authors, without undue reservation.

## Ethics statement

The studies involving humans were approved by Bambino Gesù Children’s Hospital (IRCCS) Ethical Committee. The studies were conducted in accordance with the local legislation and institutional requirements. Written informed consent for participation in this study was provided by the participants’ legal guardians/next of kin.

## Author contributions

MR: Data curation, Formal analysis, Methodology, Supervision, Validation, Visualization, Writing – original draft, Writing – review & editing, Investigation. FZ: Conceptualization, Data curation, Investigation, Methodology, Writing – original draft, Writing – review & editing. AC: Conceptualization, Data curation, Formal analysis, Resources, Validation, Writing – original draft, Writing – review & editing. EC: Conceptualization, Data curation, Methodology, Resources, Writing – original draft, Writing – review & editing. AG: Data curation, Resources, Writing – original draft, Writing – review & editing. MS: Formal analysis, Methodology, Writing – original draft, Writing – review & editing. AR: Data curation, Methodology, Writing – original draft, Writing – review & editing. CB: Data curation, Writing – original draft, Writing – review & editing. FE: Conceptualization, Data curation, Formal analysis, Investigation, Methodology, Supervision, Validation, Visualization, Writing – original draft, Writing – review & editing. MV: Conceptualization, Data curation, Formal Analysis, Funding acquisition, Investigation, Methodology, Resources, Supervision, Validation, Visualization, Writing – original draft, Writing – review & editing. MC: Conceptualization, Data curation, Formal analysis, Funding acquisition, Investigation, Methodology, Project administration, Supervision, Validation, Visualization, Writing – original draft, Writing – review & editing.
