# Supplementary material for: A novel flow cytometry panel to identify prognostic markers for steroid-sensitive forms of idiopathic nephrotic syndrome in childhood
Source: Front Immunol. 2024 Apr 2;15:1379924. doi: 10.3389/fimmu.2024.1379924 (PMC11018947; doi:10.3389/fimmu.2024.1379924)
Supplement: Supplementary file 1 [file DataSheet_1.pdf]

Supplementary Material

A novel flow cytometry panel to identify prognostic markers for steroid-sensitive forms of idiopathic nephrotic syndrome in childhood

Martina Riganati [1], Federica Zotta [2], Annalisa Candino [2], Ester Conversano [2], Antonio Gargiulo [2], Marco Scarsella [3], Anna Lo Russo [3], Chiara Bettini [2], Francesco Emma [1,2], Marina Vivarelli [1,2], Manuela Colucci [1*]

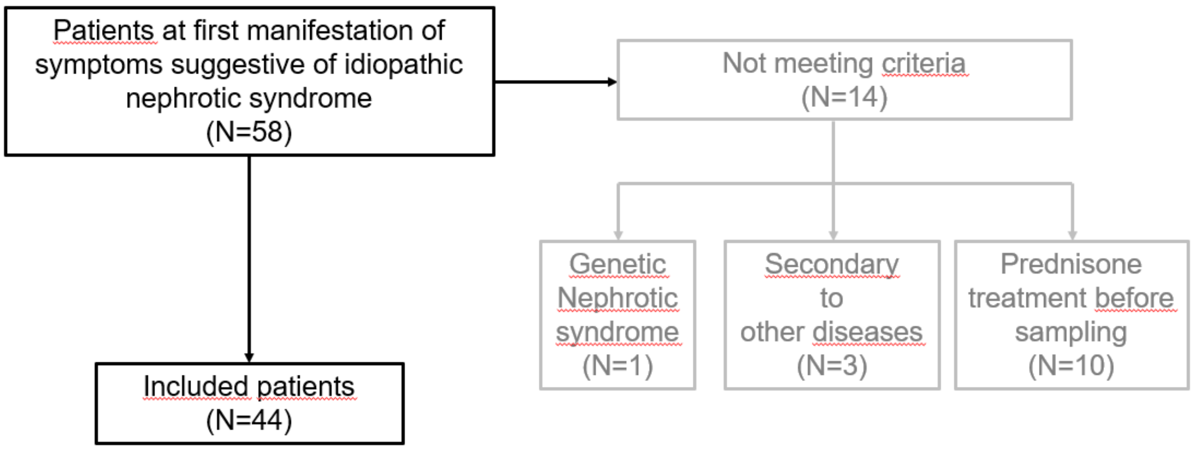

Figure S1. Patient study flowchart.

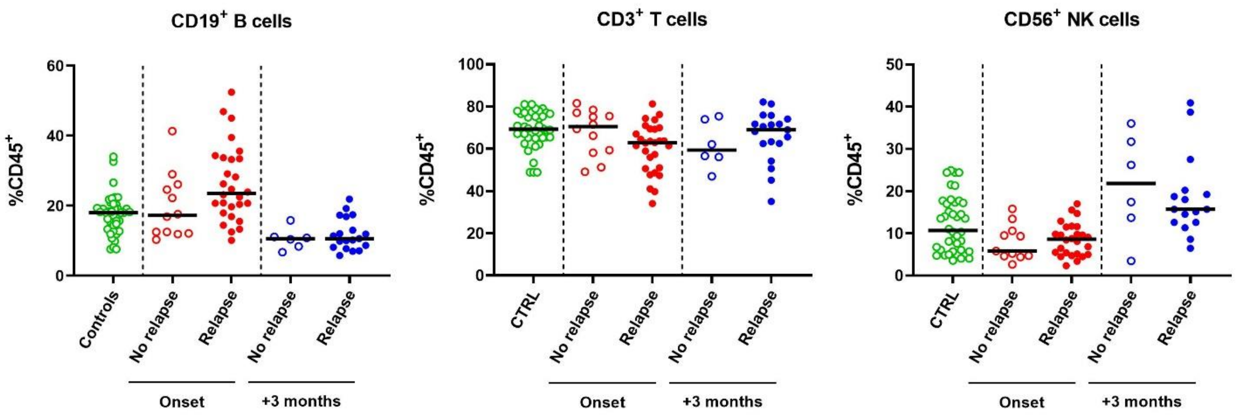

Figure S2. Levels of total B cells, T cells and NK cells of steroid-sensitive nephrotic syndrome pediatric patients who experienced or not a relapse event during a 12-month follow-up. Circulating levels of $CD19^+$ B cells, $CD3^+$ T cells and $CD56^+$ NK cells were compared between patients who relapsed or not during a 12-month follow-up as determined at onset (red dots, n=28 vs n=12) or after 3 months of prednisone therapy (blue dots, n=22 vs n=6). Age-matched controls were also represented (green dots, n=40). Lymphocyte cell subsets were expressed as percentages of total $CD45^+$ lymphocytes. Horizontal lines indicate the medians. Differences between groups were compared using unpaired Mann-Whitney U test.

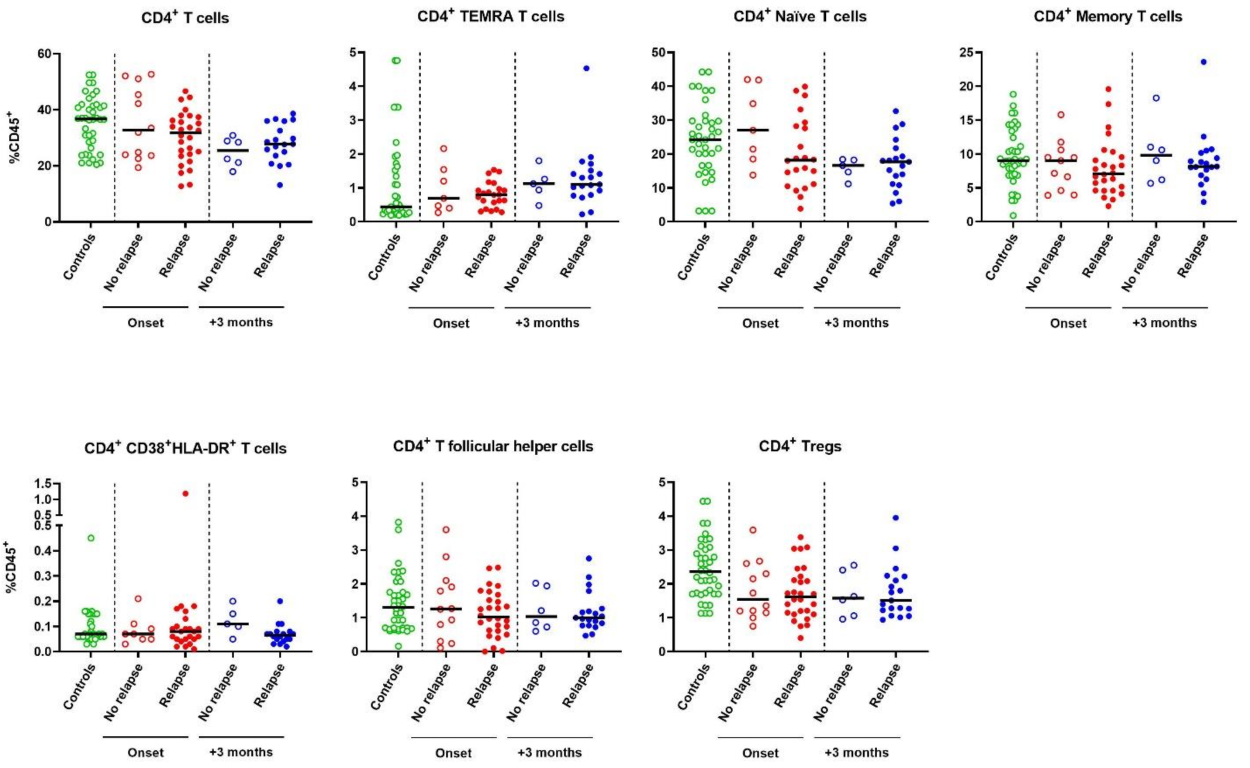

Figure S3. CD4^{+} T-cell subset profile of steroid-sensitive nephrotic syndrome pediatric patients who experienced or not a relapse event during a 12-month follow-up. Circulating levels of total CD4^{+}, CD4^{+} TEMRA, CD4^{+} Naïve, CD4^{+} memory, CD4^{+}CD38^{+}HLA-DR^{+}, CD4^{+} T follicular helper and CD4^{+} Tregs were compared between patients who relapsed or not during a 12-month follow-up as determined at onset (red dots, n=28 vs n=12) or after 3 months of prednisone therapy (blue dots, n=22 vs n=6). Age-matched controls were also represented (green dots, n=40). Lymphocyte cell subsets were expressed as percentages of total CD45^{+} lymphocytes. Horizontal lines indicate the medians. Differences between groups were compared using unpaired Mann-Whitney U test.

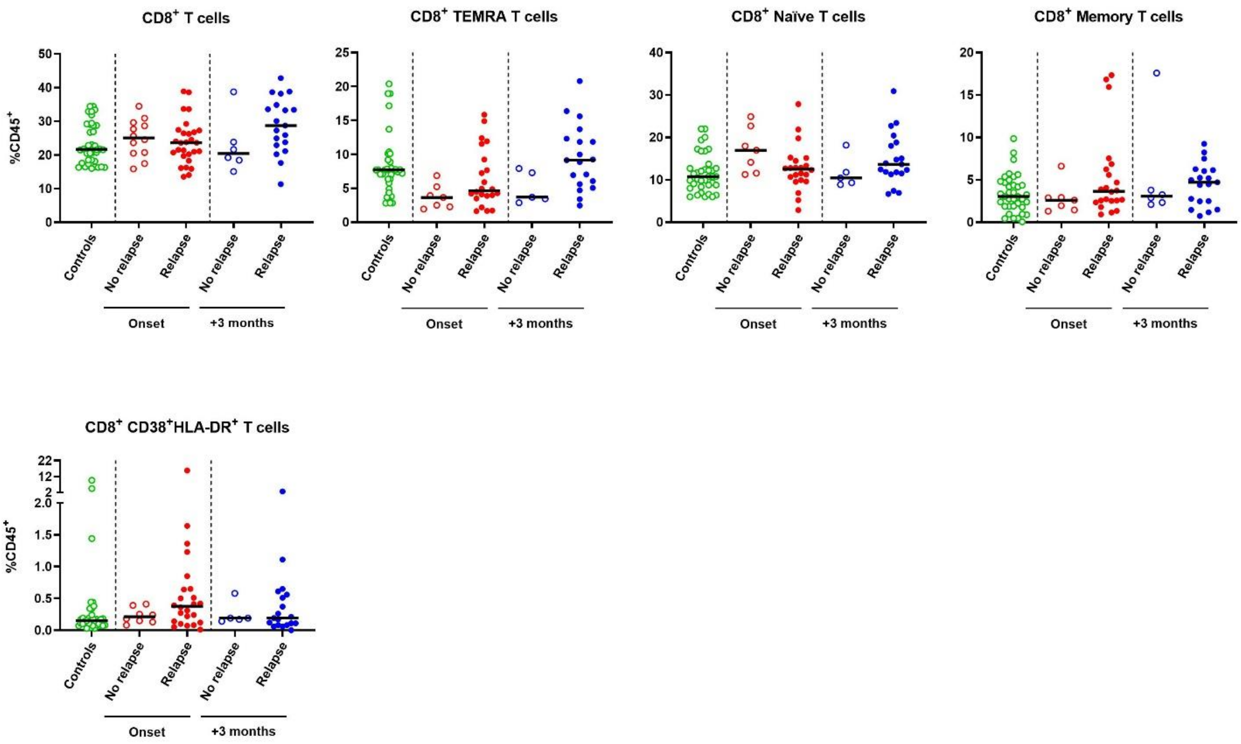

Figure S4. CD8$^+$ T-cell subset profile of steroid-sensitive nephrotic syndrome pediatric patients who experienced or not a relapse event during a 12-month follow-up. Circulating levels of total CD8$^+$, CD8$^+$ TEMRA, CD8$^+$ Naïve, CD8$^+$ memory and CD8$^+$CD38$^+$HLA-DR$^+$ T cells were compared between patients who relapsed or not during a 12-month follow-up as determined at onset (red dots, n=28 vs n=12) or after 3 months of prednisone therapy (blue dots, n=22 vs n=6). Age-matched controls were also represented (green dots, n=40). Lymphocyte cell subsets were expressed as percentages of total CD45$^+$ lymphocytes. Horizontal lines indicate the medians. Differences between groups were compared using unpaired Mann-Whitney U test.

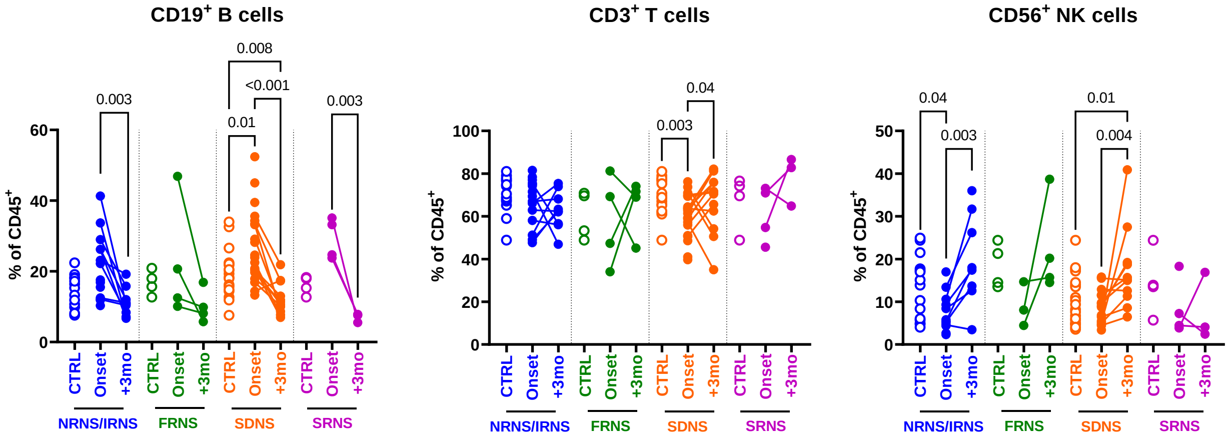

Figure S5. Levels of total B cells, T cells and NK cells of idiopathic nephrotic syndrome pediatric patients stratified based on the clinical course of their disease during a 12-month follow-up. Circulating levels of CD19$^+$ B cells, CD3$^+$ T cells and CD56$^+$ NK cells were determined in non-relapsing/infrequently relapsing (NRNS/IRNS) (n=15), frequently relapsing (n=4), steroid-dependent

(n=21) and steroid resistant (SRNS) patients at onset or after 3 months of prednisone therapy and compared to its own age-matched control subgroup. Differences between each subgroup were compared using the nonparametric Kruskal-Wallis test and, if significant, pairwise comparisons were evaluated by Dunn's test.

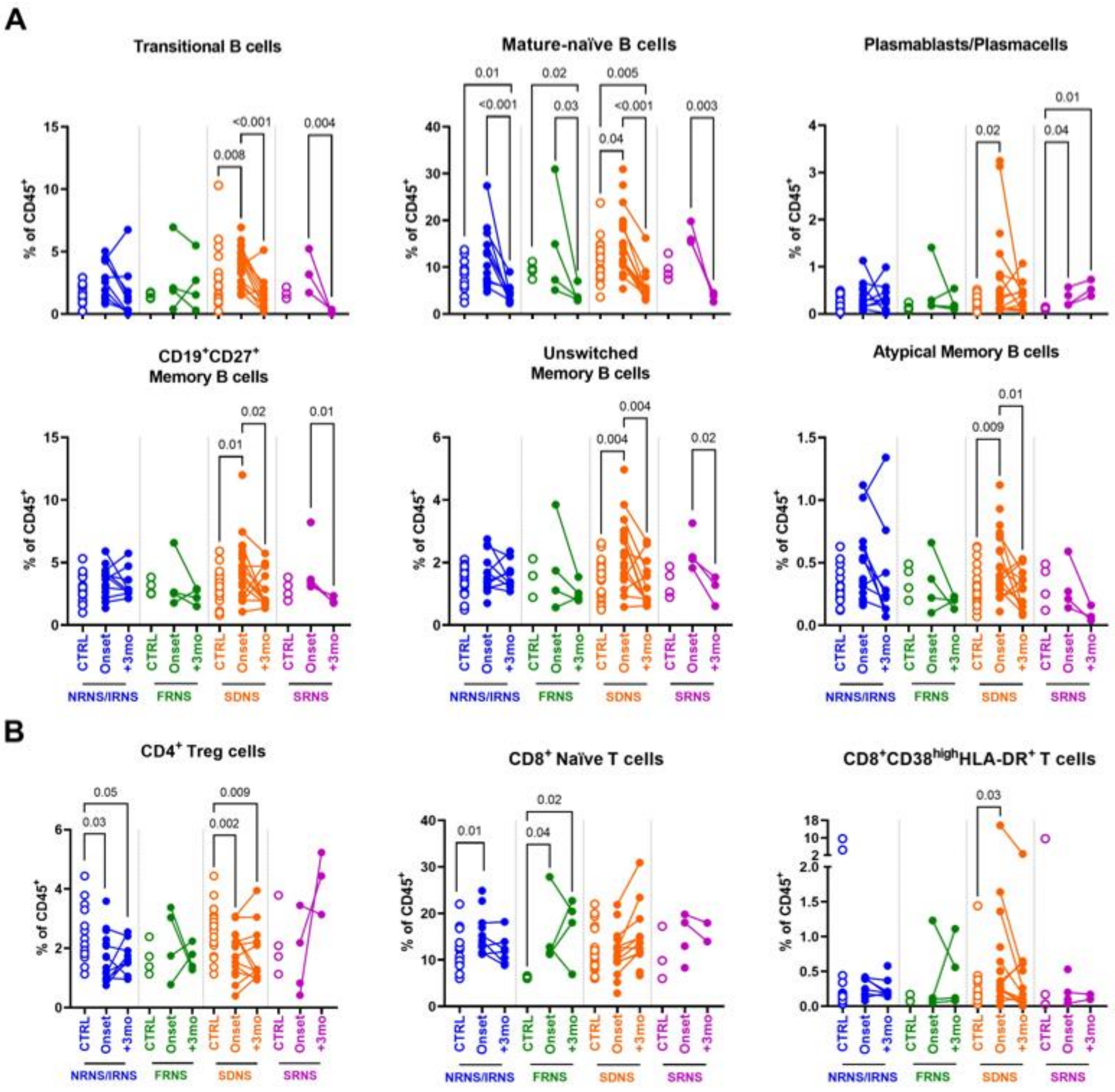

Figure S6. Levels of significantly different B-cell and T-cell subsets of idiopathic nephrotic syndrome pediatric patients stratified based on the clinical course of their disease during a 12-month follow-up compared to age-matched controls. Circulating levels of (A) transitional, mature-naïve, plasmablasts/plasmacells, $CD19^{+}CD27^{+}$ memory, unswitched memory, atypical memory and (B) $CD4^{+}$ Tregs, $CD8^{+}$ naïve and $CD8^{+}$ $CD38^{high}$ $HLA\text{-}DR^{+}$ T cells were determined in non-relapsing/infrequently relapsing (NRNS/IRNS) (n=15), frequently relapsing (n=4), steroid-dependent (n=21) and steroid resistant (SRNS) patients at onset or after 3 months of prednisone therapy and compared to its own age-matched control subgroup. Differences between each subgroup were compared using the nonparametric Kruskal-Wallis test and, if significant, pairwise comparisons were evaluated by Dunn's test.

Supplementary Table S1. Definitions relating to nephrotic syndrome in children.

**Nephrotic syndrome (NS)**	Edema, massive proteinuria (≥ 3+ on urine dipstick), hypoalbuminemia (< 3 g/dl)
**Relapse**	Proteinuria of at least 3+ for at least 3 consecutive days by urine dipstick
**Remission**	Negative or trace proteinuria for at least 3 consecutive days by urine dipstick
**Steroid-sensitive nephrotic syndrome (SSNS)**	Complete remission at 4 weeks of therapy with daily prednisone/prednisolone at standard dose.
**Infrequently relapsing nephrotic syndrome (IRNS)**	1 relapse within 6 months of initial response or ≤ 3 relapses in any 12-month period
**Frequently relapsing nephrotic syndrome (FRNS)**	≥ 2 relapses within 6 months of initial response or ≥ 4 relapses in any 12-month period
**Steroid-dependent nephrotic syndrome (SDNS)**	≥ 2 consecutive relapses during corticosteroid therapy or within 15 days of therapy discontinuation
**Steroid resistant nephrotic syndrome (SRNS)**	Lack of complete remission at 4 weeks of therapy with a standard dose of corticosteroids

TABLE S2. List of Antibodies

Marker	Fluorochrome	Clone
**Tube 1:**		
CD45	V500-C	2D1
CD19	PE-Cy7	SJ25C1
CD24	PE	ML5
CD27	APC	L128
CD38	PerCP-Cy5.5	HIT2
CD21	BV605	B-ly4
IgM	FITC	G20-127
IgG	APC-H7	G18-145
IgD	V450	IA6-2
CD3	BV786	SK7
CD56	BUV395	NCAM 16.2
**Tube 2:**		
CD3*	BV510	UCHT1
CD4*	BV786	SK3
CD8*	R718	RPA-T8
CD45RA*	PE-Cy7	HI100
CCR7*	BV711	2-L1-A
CXCR5	BB515	RF8B2
CD25	RY586	2A3
CD127	BV421	HIL-7R-M21
CD38	BUV395	HB7
HLA-DR	APC	G46-6

*These antibodies are conjugated with fluorochromes strategically designed to be complemented with defined fluorochromes with minimal resolution impact and are provided in a single kit (Human T Cell Backbone Panel Kit, BD Biosciences)

Table S3. Characteristics of patients stratified based on disease severity.

Parameters	Unit	NRNS/IRNS (n=15)	FRNS (n=4)	SDNS (n=21)	SRNS (n=4)
**Demographic**					
**Age (years)**	Years	4.8 [3.8 - 11.9]	5.45 [3.025 - 6.825]	3.4* [2.75 - 4.7]	4 [2.85 - 7.4]
**Male**	N(%)	5 (33)	4 (100)	12 (57)	1 (25)
**Clinical**					
**eGFR**	ml/min/m2	119 [111 - 156]	130 [107 - 179]	177.7** [148 - 204]	157 [106 - 210]
**serum protein**	g/dl	4 [4 - 5]	4.5 [4.0 - 5.1]	4.3 [4.1 - 4.5]	4.8 [4.6 - 5.0]
**serum albumin**	g/dl	2 [2 - 3]	2.3 [2.0 - 3.1]	2.2 [2.0 - 2.5]	3.0 [2.5 - 3.3]
**serum Cholesterol**	mg/dl	348 [256 - 463]	296 [285 - 370]	368 [316 - 416]	379 [217 - 519]
**urinary Protein/creatinine**	mg/mg	8.85 [3.91 - 18.48]	7.92 [3.05 - 26.61]	13.62 [5.23 - 23.9]	16.58 [8.87 - 28.67]
**C reactive Protein**	mg/dl	0.05 [0.03 - 0.06]	0.25 [0.03 - 1.93]	0.03 [0.03 - 0.15]	0.18 [0.03 - 0.6]
**Signs of infection**	N(%)	2 (13)	1 (25)	5 (24)	2 (50)
**Lymphocyte subsets**					
**CD19+ B cells**	% of CD45+	17.60 [12.50 - 26.20]	16.60 [10.70 - 40.35]	24.60 [20.05 - 33.85]	28.95 [24.03 - 34.63]
**CD3+ cells**	% of CD45+	69.42 [58.10 - 75.47]	58.32 [37.39 - 78.22]	61.50 [53.47 - 66.91]	62.89 [47.88 - 72.55]
**CD56+ NK cells**	% of CD45+	5.60 [4.56 - 9.78]	8.07 [4.45 - 14.70]	9.34 [5.22 - 11.58]	5.87 [4.04 - 15.54]
**Transitional B cells**	% of CD45+	1.94 [1.25 - 4.26]	1.98 [0.76 - 5.73]	3,89** [2.62 - 4.90]	3.17 [2.07 - 4.71]
**Mature-naive B cells**	% of CD45+	10.31 [6.82 - 14.77]	11.13 [5.65 - 26.94]	14,29* [11.00 - 19.41]	15,74* [15.42 - 18.83]
**Plasmablasts/plasmacells**	% of CD45+	0.26 [0.14 - 0.51]	0.24 [0.18 - 1.13]	0.44 [0.19 - 0.81]	0.30 [0.20 - 0.53]
**Atypical memory B cells**	% of CD45+	0.35 [0.21 - 0.62]	0.30 [0.13 - 0.59]	0.46 [0.31 - 0.71]	0.24 [0.16 - 0.51]
**CD19+CD27+ memory B cells**	% of CD45+	3.67 [2.26 - 4.19]	2.58 [1.97 - 5.59]	4.43 [2.83 - 5.79]	3.52 [3.16 - 7.08]
**Unswitched memory B cells**	% of CD45+	1.51 [1.22 - 2.00]	1.43 [0.71 - 3.32]	2,33* [1.53 - 2.99]	2.15 [1.90 - 2.99]
**Switched memory B cells**	% of CD45+	1.45 [0.97 - 2.17]	1.16 [0.66 - 2.29]	1.64 [0.93 - 2.69]	2.26 [0.87 - 4.50]
**IgM only memory B cells**	% of CD45+	0.21 [0.15 - 0.43]	0.21 [0.14 - 0.21]	0.19 [0.14 - 0.41]	0.15 [0.12 - 0.18]
**IgG+ switched memory B cells**	% of CD45+	0.90 [0.61 - 1.51]	0.73 [0.31 - 1.45]	1.06 [0.58 - 1.67]	0.81 [0.58 - 2.98]
**CD4+ T cells**	% of CD45+	31.93 [24.80 - 45.40]	24.43 [12.83 - 42.25]	32.59 [23.03 - 36.78]	27.85 [16.95 - 41.49]
**CD4+ TEMRA**	% of CD45+	0.81 [0.39 - 1.53]	0.82 [0.43 - 1.07]	0.73 [0.57 - 0.97]	0.88 [0.23 - 2.35]
**CD4+ Naive T cells**	% of CD45+	20.17 [15.35 - 36.61]	12.25 [7.78 - 31.80]	18.69 [14.60 - 29.32]	22.62 [12.30 - 34.13]
**CD4+ memory T cells**	% of CD45+	9.24 [6.12 - 10.89]	5.63 [3.74 - 16.21]	7.00 [4.59 - 9.07]	4.53 [1.33 - 7.69]
**CD4+CD38highHLA-DR+**	% of CD45+	0.07 [0.05 - 0.11]	0.04 [0.02 - 0.14]	0.08 [0.05 - 0.13]	0.06 [0.04 - 0.06]
**Tfh cells**	% of CD45+	1.25 [0.30 - 2.10]	0.88 [0.13 - 1.82]	0.98 [0.76 - 1.60]	0.66 [0.21 - 1.27]
**Treg cells**	% of CD45+	1.70 [1.00 - 2.30]	2.40 [1.02 - 3.30]	1.54 [1.18 - 2.17]	1.51 [0.52 - 3.14]
**CD8+ T cells**	% of CD45+	24.52 [20.72 - 28.65]	25.23 [16.53 - 31.79]	21.52 [18.96 - 27.09]	22.65 [20.16 - 24.68]
**CD8+ TEMRA**	% of CD45+	3.79 [2.45 - 7.45]	4.38 [2.32 - 6.86]	4.86 [3.89 - 11.54]	6.39 [3.76 - 8.85]
**CD8+ Naive T cells**	% of CD45+	14.67 [12.50 - 19.13]	12.13 [11.30 - 24.07]	11.15 [9.53 - 14.46]	15.49 [9.47 - 19.35]
**CD8+ memory T cells**	% of CD45+	2.58 [1.82 - 3.84]	4.09 [1.02 - 7.36]	3.72 [2.51 - 5.57]	1.50 [0.44 - 2.16]
**CD8+CD38highHLA-DR+**	% of CD45+	0.25 [0.15 - 0.41]	0.11 [0.06 - 0.95]	0.36 [0.18 - 0.75]	0.15 [0.06 - 0.45]
**Lymphocytes**	count/µl	2770 [2430 - 3420]	2550 [2250 - 4043]	3630 [2895 - 4040]	4015 [2833 - 4890]
**CD19+ B cells**	count/µl	499.5 [335.2 - 738.8]	516.6 [322.4 - 947.9]	814,5* [578 - 1202]	1186*,# [681.9 - 1695]
**CD3+ cells**	count/µl	1952 [1588 - 2152]	1444 [916.4 - 3098]	2131 [1509 - 2527]	2270 [1955 - 2671]
**CD56+ NK cells**	count/µl	165.4 [107.1 - 295.5]	198 [180.8 - 335.2]	313.5 [170.1 - 415.7]	259.1 [115.2 - 716.6]
**Transitional B cells**	count/µl	53.72 [34.47 - 97.96]	72.72 [19.84 - 141.9]	137** [79.74 - 181.8]	127.4 [60.98 - 232]
**Mature-naive B cells**	count/µl	248.1 [186.6 - 444.4]	324.3 [179.4 - 634.3]	409,2* [353.5 - 729.7]	632,7* [436.9 - 923.8]
**Plasmablasts/plasmacells**	count/µl	6.64 [3.86 - 15.34]	7.955 [5.003 - 26.08]	14.74 [5.54 - 30.75]	12.44 [5.583 - 25.68]
**Atypical memory B cells**	count/µl	8.89 [6.69 - 18.28]	10.16 [4.18 - 13.96]	15.25 [10.52 - 21.44]	9.725 [4.675 - 25.25]
**CD19+CD27+ memory B cells**	count/µl	99.75 [56.15 - 131.2]	75.28 [62.02 - 132.3]	147,5* [80.26 - 203.1]	144.7 [93.14 - 326.7]
**Unswitched memory B cells**	count/µl	42.31 [26.48 - 66.52]	37.23 [25.02 - 78.06]	87,23* [47.76 - 116.2]	82.29 [61.1 - 134.1]
**Switched memory B cells**	count/µl	40.91 [19.97 - 55.23]	36.85 [19.11 - 55.67]	66.44 [28.57 - 89.76]	100.6 [27.7 - 209]
**IgM only memory B cells**	count/µl	5.66 [3.5 - 12.21]	5.27 [3.233 - 8.26]	7.19 [3.93 - 13.05]	4.96 [4.163 - 8.398]
**IgG+ switched memory B cells**	count/µl	24.91 [12.2 - 38.71]	22.66 [9.495 - 34.38]	44.44 [16.9 - 55.1]	31.95 [18.38 - 136.8]
**CD4+ T cells**	count/µl	1027 [618.1 - 1322]	577.7 [316.1 - 1683]	1200 [762.6 - 1431]	1000 [826.2 - 1235]
**CD4+ TEMRA**	count/µl	25.89 [10.94 - 36.02]	21.87 [11.19 - 34.3]	24.78 [16.87 - 32.11]	38.13 [6.638 - 116]
**CD4+ Naive T cells**	count/µl	620 [280.1 - 1231]	300.9 [189.8 - 1331]	710 [453.9 - 1104]	764.6 [574.3 - 1079]
**CD4+ memory T cells**	count/µl	269.8 [180.1 - 329.7]	194.4 [98.45 - 396.7]	232.9 [153.9 - 321.6]	171.1 [52.98 - 244.4]
**CD4+CD38highHLA-DR+**	count/µl	2.34 [1.05 - 4.34]	1.315 [0.435 - 3.478]	2.61 [1.565 - 4.23]	1.94 [1.038 - 3.068]
**Tfh cells**	count/µl	40.43 [12.5 - 59.72]	27.62 [2.938 - 54.29]	32.36 [21.07 - 56.95]	27.08 [8.348 - 37.08]
**Treg cells**	count/µl	43.5 [27.29 - 71.14]	57.78 [26.37 - 120.4]	51.23 [31.96 - 85.43]	57.01 [25.09 - 86.86]
**CD8+ T cells**	count/µl	766.6 [511.4 - 931.9]	641.8 [374.6 - 1307]	721.3 [541.1 - 1000]	877.5 [698.6 - 1010]
**CD8+ TEMRA**	count/µl	107.2 [83.66 - 224.4]	147.3 [52.27 - 211.8]	175.8 [120.9 - 334.4]	259.6 [120 - 412.2]
**CD8+ Naive T cells**	count/µl	414.5 [286.3 - 721]	301.3 [268 - 1008]	398.1 [267.8 - 484.4]	560.2 [401.9 - 677.3]
**CD8+ memory T cells**	count/µl	72.25 [47.51 - 140.3]	106.3 [30.37 - 197.7]	115.8 [77.69 - 210.1]	44.26 [17.28 - 89.89]
**CD8+CD38highHLA-DR+**	count/µl	6.52 [4.91 - 10.22]	4.07 [1.69 - 21.75]	12.16 [4.92 - 26.69]	5.12 [2.37 - 19.31]

*, **, significant differences vs NRNS/IRNS

#, significant difference vs FRNS
